# Liposarcome dédifférencie du cordon spermatique: difficultés thérapeutiques des grosses tumeurs

**DOI:** 10.4314/pamj.v8i1.71167

**Published:** 2011-04-28

**Authors:** Ghizlane Rais, Marwane Benatiya Andaloussi, Soundouss Raissouni, Ali Barki, Mohamed Allaoui, Fouad Zouaidia, Mohamed Afif, Hind Mrabti, Hassan Errihani

**Affiliations:** 1 Service d’oncologie médicale, Institut National d’oncologie, Rabat, Maroc; 2 Service d’Urologie A CHU Ibn Sina, Rabat, Maroc; 3 Service d’anatomopathologie, CHU Ibn Sina, Rabat, Maroc; 4 Service de radiothérapie, Institut National d’oncologie, Rabat, Maroc

**Keywords:** Liposarcome, cordon spermatique, Chirurgie, radiothérapie

## Abstract

Le liposarcome du cordon spermatique est une entité rare : environ 100 cas ont été rapportés dans la littérature. Nous rapportons l’observation d’un homme âgé de 42 ans, chez qui a été décelée une masse tumorale développée aux dépens du cordon spermatique droit. Une orchidectomie droite avec exérèse large de la tumeur a été difficilement réalisée en raison de la taille importante de la masse. En post opératoire, le patient a présenté une progression locale et métastatique pulmonaire. Une mono chimiothérapie a été administrée à base d’anthracycline mais le patient a décédé suite à une progression rapide de la maladie. À travers cette observation, nous rapportons brièvement les données de la littérature de cette entité rare. Une exérèse large avec des marges saines, tant que possible, est indispensable pour le contrôle local de la maladie. Néanmoins, en cas de taille tumoral importante, comme le cas de notre patient, une exérèse complète est souvent difficile. Vu le taux élevé de rechute locale, une stratégie combinée associant chirurgie et radiothérapie adjuvante peut être envisagée. Le rôle de la chimiothérapie, bien qu’incertain, garde son indication dans les cas métastatiques, surtout dans les sous types dédifférenciés.

## Introduction

Le liposarcome paratesticulaire est une tumeur rare, survenant essentiellement chez le sujet âgé [1]. Son développement à partir du cordon spermatique est exceptionnel, avec aproximativement 100 cas rapportés dans la littérature [1,2]. Il est difficile par conséquent d’accumuler suffisamment d’observations, d’une part, pour établir une corrélation entre le grade histologique et le pronostic et, d’autre part, pour démontrer un quelconque bénéfice d’un traitement complémentaire après la chirurgie. La littérature relative à sa prise en charge est limitée dans la plupart du temps à des petites séries ou à des rapports de cas cliniques. Nous rapportons le cas d’un patient de 42 ans, chez qui a été diagnostiqué un liposarcome dédifférencié du cordon spermatique, de taille importante et rapidement évolutive posant le problème d’une exérèse complète, avec rechute postopératoire immédiate locorégionale et métastatique.

## Observation

**Figure 1:**
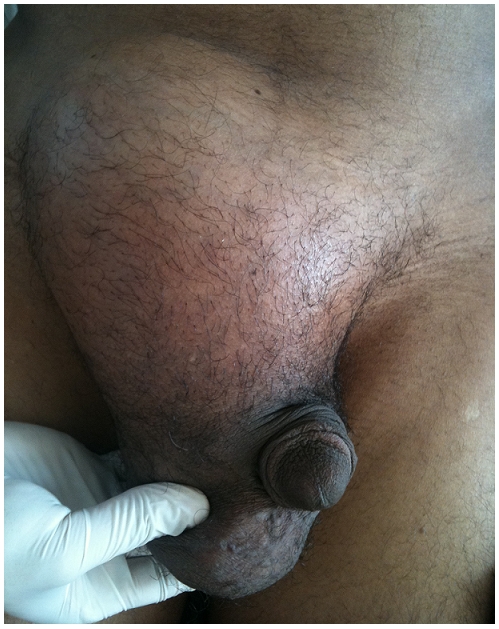
Image clinique d’une volumineuse masse inguinale droite chez un patient de 42 ans admis au service d’urologie pour prise en charge d’une masse tumorale développée aux dépens du cordon spermatique droit.

Un homme de 42 ans a été admis au service d’urologie pour prise en charge d’une masse tumorale développée aux dépens du cordon spermatique droit. Le patient n’avait pas d’antécédents médicochirurgicaux particuliers. L’histoire de sa maladie remonte à 5 mois par la découverte d’une masse paratesticulaire augmentant progressivement de volume. L’examen clinique mettait en évidence la présence d’une masse inguinale droite de 15 cm de grand axe, de consistance molle avec des signes inflammatoires en regard et sensible à la palpation (Figure 1).

**Figure 2:**
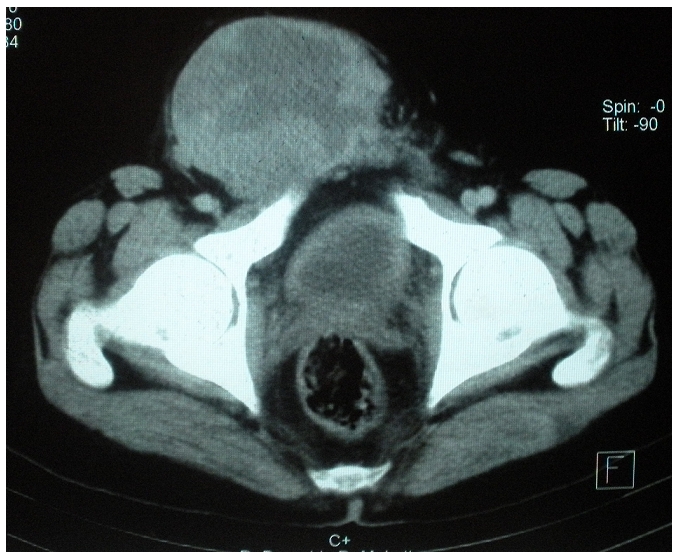
Image tomodensitométrique inguinoscrotal montrant une grande masse s’étendant vers le canal inguinal droit chez un patient de 42 ans admis au service d’urologie pour prise en charge d’une masse tumorale développée aux dépens du cordon spermatique droit.

L’examen du scrotum retrouve des testicules palpables d’aspect normal. Les aires ganglionnaires périphériques étaient libres et l’examen abdominal était normal. Les marqueurs tumoraux (alphafœto -proteine, Béta-HCG, ACE) étaient normaux. La tomodensitométrie inguino-scrotale mettait en évidence une volumineuse masse scrotale droite s’étendant dans le canal inguinal jusqu’au niveau de la fosse iliaque droite (Figure 2). La radiographie pulmonaire, la TDM thoraco- abdominale étaient normales. Une orchidectomie droite par voie inguinale et une exérèse large de la tumeur a été difficilement réalisée à cause de la taille importante de la tumeur. L’examen macroscopique de la pièce opératoire mettait en évidence un testicule normal avec une albuginée intacte refoulé par une masse ferme, faisant 15x10x8 cm, homogène, de consistance molle, d’aspect jaunâtre, et infiltrant complètement le cordon spermatique.

**Figure 3:**
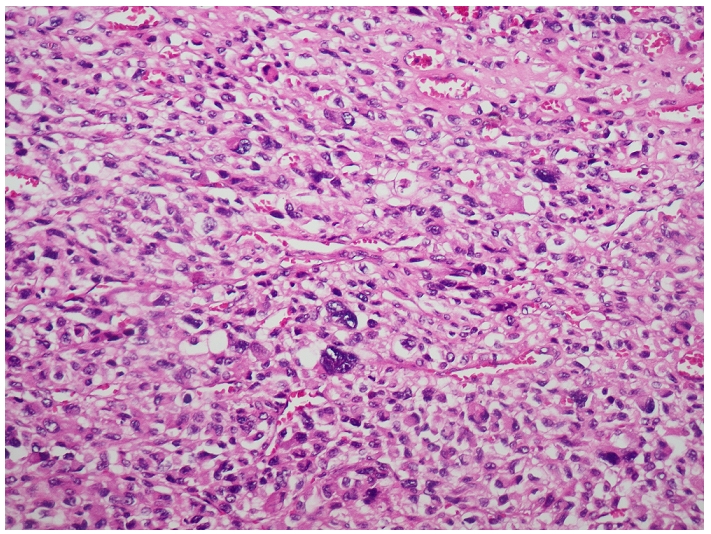
Les cellules tumorales sont pléomorphes avec présence de monstruosités nucléaires (HE, Gx400) chez un patient de 42 ans avec diagnostic de liposarcome dédifférencié.

**Figure 4:**
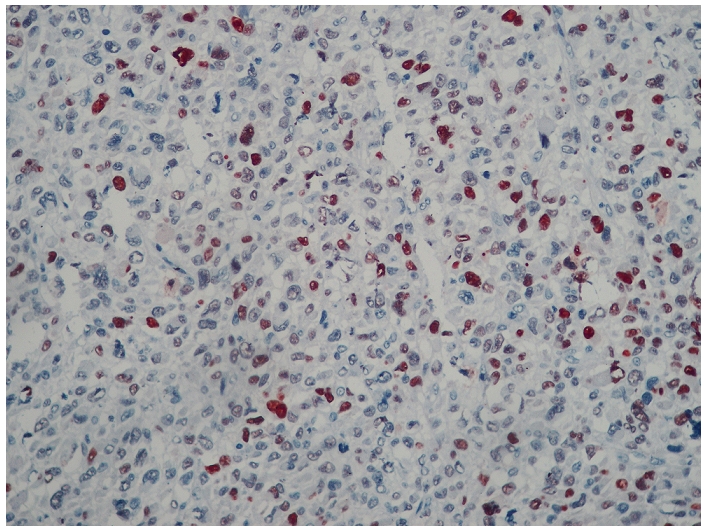
Immunomarquage montrant l’expression du MDM2 par les cellules tumorales (Gx400) chez un patient de 42 ans avec diagnostic de liposarcome dédifférencié

L’étude microscopique notait la présence d’une prolifération tumorale de densité cellulaire élevée (Figure 3) et faite d’adipocytes pléomorphes aux noyaux très irréguliers. Certains de ces noyaux avaient des atypies marquées avec focalement la présence de cellules monstrueuses plurinucléées. L’analyse immunohistochimique a montré l´expression du MDM2 (Figure 4) par les cellules tumorales alors que les Ac anti myogénine et anti desmine étaient négatifs. Devant ces aspects morphologiques et immunohistochimiques, le diagnostic de liposarcome dédifférencié a été posé. L’évolution a été marquée par une récidive locorégionale deux mois après la chirurgie Le bilan scannographique d’extension mettait en évidence une récidive locale associée à des localisations métastatiques pulmonaires multiples en « lâcher de ballon ». Après un bilan de tolérance sans anomalies, le patient a été mis sous chimiothérapie palliative à base d’anthracycline à la dose de 75mg/m^2^ tous les 21jours. Le patient a décédé au décours de la troisième cure suite à une progression franche locorégionale et métastatique.

## Discussion

Les tumeurs malignes paratesticulaires sont rares, comprenant principalement des sarcomes provenant du mésenchyme du cordon spermatique. Le premier cas de sarcome du cordon a été signalé par Lesauvage en 1845 [1-3]. Depuis, approximativement 100 cas de liposarcome du cordon spermatique ont été signalés dans la littérature mondiale [4]. Les liposarcomes sont de loin les sarcomes les plus fréquents de la région paratesticulaire (4à 20%) [5-7]. Ils sont sous diagnostiqués et forment un spectre de lésions d’agressivité variable. Les plus grandes séries publiées proviennent de grands centres de cancérologie aux États-Unis. À notre connaissance, la plus grande série (et la seule prospective) est celle de Coleman et al. [8], avec 47 cas rapportés de 1982 à 2001. En raison du nombre limité de cas de liposarcome spermatique rapportés, les meilleures approches thérapeutiques et pronostiques ne sont pas bien élucidées.

Bien que rares, cette tumeur affecte les sujets adultes de tout âge, généralement de la cinquantaine ou soixantaine, avec une tranche d´âge entre 16 et 88 ans [9,10]. Il n´y a qu´une seule observation rapportée d´un patient de 6 ans [11].

Certains auteurs estiment que la lésion survient à partir des tissus du cordon spermatique; d´autres croient qu´elle peut survenir à partir d’une dégénérescence maligne de lipome préexistant [6]. Il a également eu des spéculations sur le rôle joué par des facteurs tels que les traumatismes locaux (plusieurs interventions chirurgicales). En effet, l´histoire naturelle du liposarcome du cordon spermatique n´est pas bien comprise, même si pour d´autres liposarcomes il ya souvent l´histoire sous-jacente de lésions ressemblant aux lipomes à évolution lente, qui se mettent soudain à augmenter de taille [6].

Cliniquement, le liposarcome du cordon spermatique se présente sous forme d’une masse nodulaire ferme, de croissance lente, indolore, de taille variable (3-30cm) et peut atteindre des proportions gigantesques (jusqu´à 13,5 kg) comme le cas de notre patient. Elle est généralement située au-dessus du testicule en intrascrotal ou au niveau de l´aine. La présentation décrite ici est assez atypique. La masse est plutôt inguinale que scrotale. Le diagnostic clinique est souvent difficile posant le problème de diagnostic différentiel avec la hernie inguinale, l’hydrocèle, l’épididymite chronique et plus fréquemment avec le lipome, ce qui peut engendrer des retards diagnostiques. Néanmoins, La croissance rapide, la taille importante et la présence de symptômes sont des critères en faveur de la malignité [12]. Sur le plan radiologique il n’existe pas de signes radiologiques caractéristiques. La tomodensitométrie et l’IRM ne semblent pas supérieures à l’échographie dans l’exploration locale des tumeurs du cordon spermatique [1,13]. L’Échographie inguino-scrotale met en évidence typiquement des lésions solides, hyperéchogènes et hétérogènes mais celles-ci peuvent se présenter comme des nodules indurés de petite taille au sein d’un tissu adipeux de consistance proche de la normale, ne permettant pas ainsi la distinction entre les lésions bénignes et malignes.

La tomodensitométrie apporte la preuve de la localisation, de l´étendue, et de la relation de la masse intrascrotale avec le testicule, l’épididyme, et le cordon spermatique. Les liposarcomes apparaissent comme des masses graisseuses, comme ils peuvent être hypodenses par rapport à la graisse sous cutanée [8,14]. FDG-PET scan peut être utile dans les cas récurrents mais son utilisation en routine n´est pas indiquée [15,16].

Il n’y a pas de corrélation entre le volume de la tumeur et sa malignité. Le caractère limité, indolore peut être faussement rassurant, et souvent le diagnostic n’est pas affirmé en pré-opératoire. Seule l’intervention chirurgicale effectuée, soit pour une pathologie différente, soit devant la suspicion d’une tumeur du cordon ou plus souvent d’une tumeur «intra-scrotale», permet de faire le diagnostic.

Sur le plan histologique, la classification de l´organisation mondiale de la Santé OMS des tumeurs des tissus mous reconnaît cinq sous types de liposarcomes qu’on peut répartir en trois entités distinctes différentes dans leur localisation, leur épidémiologie, leur clinique et leur imagerie : a. le liposarcome bien différencié/dédifférencié b. liposarcome myxoïde ou à cellules rondes, etc. Le liposarcome pléomorphe [17-19].

**Le liposarcome bien différencié** : ou lipoma-like est caractérisé par une prolifération d’adipocytes matures de tailles et de formes variables, associées à des cellules stromales atypiques fusiformes au noyau hyperchromatique prédominant dans les septa fibreux.

**Le liposarcome indifférencié** associe des zones de liposarcome bien différenciées et d’autres pauvrement différenciées.

Ces deux entités sont caractérisées par des anomalies génétiques particulières sur le chromosome 12, dont l’amplification des gènes MDM2/CDK4 [19].

**Le liposarcome myxoïde** constitué de cellules fusiformes dans une matrice myxoïde, abondante, peu cellulaire, caractérisée par un riche réseau capillaire, grêle.

**Le liposarcome à cellules rondes** composé de nappes homogènes de cellules rondes, ovales ou parfois fusiformes relativement régulières.

Ces deux derniers types histologiques sont caractérisés génétiquement par une translocation simple t (12;16) et t(12;22).

**Le liposarcome pléomorphe** riche en cellules aux noyaux très atypiques, nucléolés et souvent en mitose, de caryotype complexe et métastasant au niveau des ganglions régionaux.

Le diagnostic différentiel d’un lipome avec un liposarcome bien différencié peut être difficile : la surexpression à l’immunohistochimie de MDM2 et/ou CDK4 permet d’établir le diagnostic, même si cette surexpression n’est cependant ni sensible (MDM2) ni spécifique (CDK4) à 100%, rendant l’appréciation des marges chirurgicales délicates [20].

L’agressivité des liposarcomes est fonction du volume des secteurs dédifférenciés. La dédifférenciation peut survenir de novo ou au cours de récidives itératives d’un liposarcome bien différencié ce qui implique bien l’importance d’une exérèse complète.

Suivant les principes généraux du traitement du sarcome, l’orchidectomie radicale et la résection large de la tumeur avec des marges négatives microscopiques sont essentiels dans la gestion du sarcome du cordon spermatique. Toutefois, ces sarcomes se produisant dans cette région anatomique, se prêtent rarement à l´excision large, et les marges de résection sont presque toujours envahies. Ainsi, la récidive locale est un problème majeur. La plupart des séries ont rapporté des taux de récidive locale d’approximativement 50% après la chirurgie lorsque ce traitement était unique [8,21].

Par exemple, dans une série de 47 patients atteints de sarcomes du cordon spermatique principalement liposarcomes, Coleman et al. [8] ont rapporté une survie sans maladie plus courte avec des marges chirurgicales positives.

Il est bien établi pour les sarcomes des tissus mous que le grade de la tumeur a peu d´influence sur la probabilité de récidive locale. L´influence de la taille de la tumeur sur la probabilité de récidive locale est également incertaine. Néanmoins, pour les sarcomes survenant dans les sites où les contraintes anatomiques limitent la résection adéquate, on s´attend à ce que les tumeurs de grande taille rechutent plus fréquemment que les petits sarcomes. En résumé, tous les types de sarcomes du cordon spermatique, quelque soit leur grade et leur taille dans ont une tendance significative à la récidive locale, lorsqu´ils sont gérés par la chirurgie seule. Ce taux élevé de récidive locale indépendamment du type histologique et de la taille tumorale pose la question de l’opportunité d’un traitement adjuvant.

Le rôle de la radiothérapie est encore incertain. Alors que Coleman [8] (47 patients) a rapporté que la radiothérapie adjuvante ne diminue pas significativement le taux de récidives locales et n’améliorait pas la survie globale, 3 autres auteurs [21-23] ont noté un contrôle plus durable a été observée après la chirurgie et la radiothérapie combinée. Selon ces auteurs, un traitement combiné est une modalité qui devrait être envisagée.

Le rôle de la chimiothérapie est également incertain. La plupart des études sont des rapports de cas ou des séries d´un petit nombre de patients. Pour les patients atteints de la maladie métastatique non résécable, une utilisation judicieuse de la chimiothérapie offre une palliation des symptômes et évite une progression rapide de la maladie. Le choix du protocole de chimiothérapie est souvent calqué sur ceux de liposarcome des tissus mous.

Les liposarcomes myxoïdes et à cellules rondes sont parmi les sous-types les plus sensibles à la chimiothérapie Les sous types dédifférenciées présentent une grande variabilité individuelle de chimiosensibilité [24]. La chimiothérapie la plus utilisée dans la littérature pour cette localisation rare est l’anthracycline à des doses de 60 à 80mg/m^2^ [8,17,23].

## Conclusion

Le cordon spermatique représente une localisation rare de liposarcome, dont les aspects morphologique et immunophénotypique sont identiques à ceux décrits dans d’autres localisations. La stratégie la plus probable de réduire ce taux de récidive locale élevé serait une combinaison de chirurgie et de radiothérapie. Néanmoins, aucune série n’est pas suffisamment importante pour évaluer définitivement l´efficacité de cette stratégie, et des études prospectives sont exclues du fait de la rareté de cette maladie.

## Conflit d’intérêts

Les auteurs ne déclarent aucun conflit d’intérêts.

## Contributions des auteurs

Tous les auteurs ont contribué à la rédaction de ce manuscrit et lu et approuvé la version finale.
